# Causes of death among persons of all ages within the Kilifi Health and Demographic Surveillance System, Kenya, determined from verbal autopsies interpreted using the InterVA-4 model

**DOI:** 10.3402/gha.v7.25593

**Published:** 2014-10-29

**Authors:** Carolyne Ndila, Evasius Bauni, George Mochamah, Vysaul Nyirongo, Alex Makazi, Patrick Kosgei, Benjamin Tsofa, Gideon Nyutu, Anthony Etyang, Peter Byass, Thomas N. Williams

**Affiliations:** 1 KEMRI-Wellcome Trust Research Programme, Kilifi, Kenya; 2 INDEPTH Network of Demographic Surveillance Sites, Accra, Ghana; 3 United Nation Statistics Division, New York, NY, USA; 4 Umeå Centre for Global Health Research, Department of Public Health and Clinical Medicine, Umea University, Umeå, Sweden; 5 Department of Medicine, Imperial College, London, UK

**Keywords:** verbal autopsy, InterVA-4, cause-specific mortality fraction, Kenya

## Abstract

**Background:**

The vast majority of deaths in the Kilifi study area are not recorded through official systems of vital registration. As a result, few data are available regarding causes of death in this population.

**Objective:**

To describe the causes of death (CODs) among residents of all ages within the Kilifi Health and Demographic Surveillance System (KHDSS) on the coast of Kenya.

**Design:**

Verbal autopsies (VAs) were conducted using the 2007 World Health Organization (WHO) standard VA questionnaires, and VA data further transformed to align with the 2012 WHO VA instrument. CODs were then determined using the InterVA-4 computer-based probabilistic model.

**Results:**

Five thousand one hundred and eighty seven deaths were recorded between January 2008 and December 2011. VA interviews were completed for 4,460 (86%) deaths. Neonatal pneumonia and birth asphyxia were the main CODs in neonates; pneumonia and malaria were the main CODs among infants and children aged 1–4, respectively, while HIV/AIDS was the main COD for adult women of reproductive age. Road traffic accidents were more commonly observed among men than women. Stroke and neoplasms were common CODs among the elderly over the age of 65.

**Conclusions:**

We have established the main CODs among people of all ages within the area served by the KHDSS on the coast of Kenya using the 2007 WHO VA questionnaire coded using InterVA-4. We hope that our data will allow local health planners to estimate the burden of various diseases and to allocate their limited resources more appropriately.

In developed countries, cause-specific mortality data are readily available from well-established vital registration systems. In developing countries, however, where a majority of the world’s deaths occur, vital registration systems are poor (1), access to health care services is limited, and most deaths occur at home. Reliable medical records on causes of death (CODs) are inadequate, and therefore other sources of data must be used to investigate patterns of cause-specific mortality.

Despite the fact that some studies have questioned the reliability of the Verbal Autopsy (VA) approach, where close relatives of the deceased are asked about the signs and symptoms that precede deaths (2, 3), it remains the only practical approach through which to estimate CODs in most resource-poor settings in which the majority of deaths occur outside the formal health system (2, 4–8) . The approach has been used to determine CODs among people of all ages in a wide range of settings (2, 7, 9–16); however, in Kenya, although VAs have been widely used as a tool for documenting CODs (7, 12–14, 17–21) , no study has yet been published in which the WHO-compliant InterVA-4 has been applied across deaths of all ages. The InterVA-4 model uses posterior probabilities for CODs, given an a priori distribution of CODs in the population and conditional probabilities for circumstances leading to death. The current study was conducted with a view to using the InterVA-4 model to describe CODs among all residents of the area served by the Kilifi Health and Demographic Surveillance System (KHDSS), where close to 60% of deaths occur outside the formal health system. This study is also part of the multisite cause of death dataset (22) available in the public domain at the INDEPTH Data Repository (23).

## Methods

### Study population

The KHDSS study area has been described in detail previously (24). Briefly, the KHDSS was established in 2000 and became a member of INDEPTH network in 2005. The KHDSS covers an area of 891 km^2^ and has a current resident population of ~265,000. The population register is updated three times each year and records between 1,200 and 1,500 deaths annually.

VA, using the standard 2007 World Health Organization (WHO) VA tools (25), were first introduced into the KHDSS activities in 2008 (12). These tools include three separate VA questionnaires that are used to collect data on neonates (0–28 days old), children (29 days to 14 years old), and adolescents and adults (>15 years). Structurally, the questionnaires contain a short open narrative section that is followed by a cascade of closed questions. The narrative part provides the respondent’s chronological account of any illnesses and events that led to death, while the closed questions filter through the history and details of the illness. Age-appropriate VA questionnaires were generated for each death along with household maps and listings to guide interviewers to the appropriate respondents. With a view to minimizing transcription errors, we generated pre-filled VA questionnaires that included all basic demographic details such as names, personal identifiers, dates of birth and death for confirmation by the interviewers before their interviews. Completed VA forms were checked for errors by the field supervisor before entry into a database written in FileMaker Pro™ v11 (FileMaker, USA).

### The InterVA-4 model

CODs were assigned using the InterVA-4 computer-based probabilistic model (25). The original model used an expert panel to develop a coding system based on relatively broad COD categories. The current version (Version 4.02) (25) has evolved, and has benefitted from testing and validation, largely using data from sub-Saharan Africa (4, 12, 13). The InterVA-4 software is freely available in the public domain (25). For our analysis, COD categories were obtained by running InterVA-4 in batch mode on the input indicators with both malaria and HIV prevalence set to ‘high’ for all the age categories.

### Data management

All VA forms were scrutinized by a clinician for anomalies before double entry into a computer database written in FileMaker Pro™ v11 (FileMaker, USA). The VA data were further transformed to align with the WHO 2012 VA standard format and were then processed using the InterVA-4 model to assign COD. These VA standard formats and the specifications have been described in detail previously (26). Where available, we supplemented the model with information from the open narrative section for input data variables that were not targeted systematically in the closed section of the VA questionnaires. Of particular note, we included data on a set of reported hospital diagnoses that were required as input data for InterVA-4 but that were not included on the list of previously known medical conditions in the 2007 WHO questionnaires. Examples included sickle cell disease (SCD) and congenital malformations. Such information was extracted by keyword searches of the free-text narrative section. For example, to create the congenital malformation (born_malf) indicator, we searched for the strings ‘heart’, ‘congenital’, ‘hydrocephalous’, ‘spina bifida’, and ‘hole in the heart’ within VAs collected from children who died at <5 years of age. The congenital malformation indicator was left unchanged if it had already been marked ‘YES’ but was converted from ‘NO’ to ‘YES’ in cases where heart disease had been selected in the list of previously known medical conditions. Similarly, injuries associated with sexual assault (rape) were not always captured in the structured questions but were sometimes described in the narrative section, facilitating mapping to the ‘assault’ indicator. Injury may either be intentional (e.g. assault or suicide) or accidental. The main warning messages flagged by the model when we input our data, involved cases where, for example, suicide was inconsistent with other injuries.

Finally, the variable ‘born_small’ requires either the birth weight or data to suggest that the child was small at birth as reported by the respondent. Some respondents indicated that the baby was born small while the birth weight recorded was more >2.5 kg or vice versa. Where responses were recorded for both indicators, therefore, we only considered the birth weight in mapping the ‘low birth weight’ (born_small) or ‘big baby’ (born_big) indicators.

### Data analysis

Deaths were aggregated for all individuals in the study population for the period January 2008 to December 2011. An analytical dataset was constructed from the model’s output in which each VA case had one or more records. Each record having one cause and a weight corresponding to the likelihood of that cause for the particular VA case. The model assigned multiple CODs if they reached half of the likelihood of the leading cause. Any residual margin of likelihood not accounted for by the likelihood of the first, second or third causes was then considered as indeterminate. The possible CODs determined by the InterVA-4 model were derived for all ages. Cause-specific mortality fractions (CSMF) were determined as the proportion of all deaths that were attributable to any specific COD. We stratified mortality analyses by sex and seven age groups (neonates, infants, and ages 1–4, 5–14, 15–49, 50–64 and over 65 years). The age-group boundaries were chosen to reflect groups of public health importance. All statistical analyses were carried out using STATA V11 (Timberlake, USA) and the statistical software environment R (http://www.r-project.org/).

### Ethical approval

Individual written informed consent was obtained by interviewers from all VA respondents. The study was approved by the KEMRI/Wellcome Trust Scientific Coordinating Committee (SCC) and by the KEMRI Scientific Steering Committee (SSC), both in Kilifi, and by the KEMRI/National Ethical Review Committee (ERC) in Nairobi.

## Results

A total of 5,187 deaths were recorded among the resident population of the KHDSS between January 2008 and December 2011. One thousand one hundred and eighty one cases (23%) were among children <5 years old, 242 cases (5%) were aged 5–14 years, and 3,764 cases (73%) were >15 years of age, of which 1,665 cases (32%) were >65 years old (Table 1). Of the deaths among children <5 years old, 810 (68%) occurred before the first year of life of which 469 (58%) were neonates. Male deaths out-numbered female deaths in most age groups. VA interviews were successfully completed for 4,460 (85%) of these deaths of which 2,304 (52%) were among males (Table 1). Failure to identify an appropriate respondent was the main reason for missing data. Among those deaths in which a VA was performed, 57% occurred at home and 36% occurred in a health facility. Most neonatal deaths occurred in hospitals while more of the elderly died at home. The average recall period (time between VA interview and death) was 194 days. Among those aged >15 years (*n=*3,310), 10% were single, 55% were legally married, 28% were widowed, 4% were divorced, and 3% were separated. The InterVA-4 model assigned a single COD to 3,886 cases (87%), two CODs to 422 cases (9%), three CODs to 22 cases (1%), and 130 cases (3%) to ‘indeterminate’.

**Table 1:** Total number of deaths and percentages with VA by age group and sex

	Age group															
	
Sex	Neonate		Infant		1–4 years		5–14 years		15–49 years		50–64 years		65+ years		Total	
	Deaths	VA	Deaths	VA	Deaths	VA	Deaths	VA	Deaths	VA	Deaths	VA	Deaths	VA	Deaths	VA
Females	209	70	162	89	166	79	103	88	687	82	408	85	818	89	2,553	84
Males	260	76	179	79	205	83	139	91	569	88	435	88	847	92	2,634	87
Total	469	73	341	84	371	81	242	90	1256	85	843	86	1665	91	5,187	86

Deaths: total deaths within the study area; VA: percentage of deaths for which a verbal autopsy was conducted.

### Cause-specific mortality fractions

The top five CODs for the whole population overall were: HIV-/AIDS-related causes, acute respiratory infections including pneumonia, malaria, and pulmonary tuberculosis. Pneumonia was the most common COD among the infants while malaria and HIV were the commonest CODs among children 1–4 years and adults aged 15–49 years, respectively. Pulmonary tuberculosis was the leading COD among the elderly age groups.

#### Neonatal deaths (*n*=344)

**Fig. 1:**
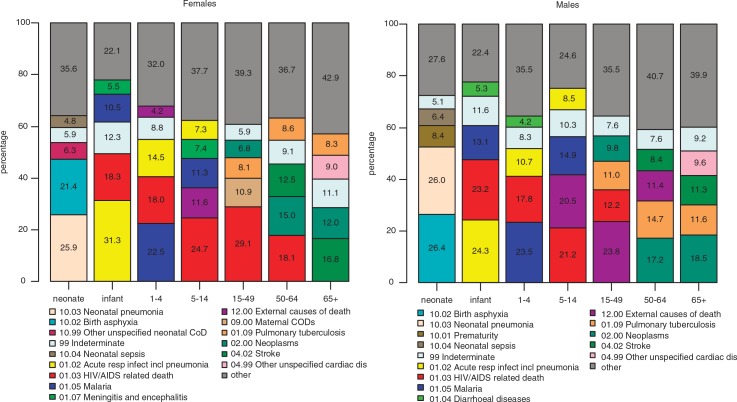
Cause-specific mortality fractions by age group and sex. The figure shows the top five cause-specific mortality fractions among each age group by sex as derived by the InterVA-4 model.

The majority (78%) of neonatal deaths occurred in the first 7 days of life: 31% occurred within the first 24 hours, and 47% between 2 and 6 days of age. The leading CODs in neonates were pneumonia (26%), birth asphyxia (24%), prematurity (6%), and sepsis (6%) (Table 2). Five percent of deaths were indeterminate. Death due to prematurity was more common among males than females (Fig. 1).

**Table 2:** Cause-specific mortality fractions by age group as assigned by the InterVA-4 model

Cause of death^a^	Neonates	Infants	1–4 years	5–14 years	15–49 years	50–64 years	65+ years
01.01 Sepsis (non-obstetric)		1	0	0	0	0	0
01.02 Acute resp infect incl. pneumonia		28	12	8	3	3	5
01.03 HIV-/AIDS-related death		21	18	23	21	12	3
01.04 Diarrheal diseases		4	3	1	1	1	1
01.05 Malaria		12	23	13	1	0	1
01.06 Measles		2	1	0			
01.07 Meningitis and encephalitis	1	4	1	5	2	0	0
01.08 & 10.05 Tetanus						0	
01.09 Pulmonary tuberculosis			0	1	9	12	10
01.11 Hemorrhagic fever			0		0		
01.99 Other and unspecified infect dis			0	1	0	0	0
02.01 Oral neoplasms					0		0
02.02 Digestive neoplasms					2	5	3
02.03 Respiratory neoplasms					3	8	6
02.04 Breast neoplasms					0		
02.05 & 02.06 Reproductive neoplasms MF					1	1	1
02.99 Other and unspecified neoplasms					2	3	4
03.01 Severe anemia					0		0
03.02 Severe malnutrition		1	2	1	1	2	2
03.03 Diabetes mellitus			0		0	1	2
04.01 Acute cardiac disease					1	1	1
04.02 Stroke					3	10	14
04.03 Sickle cell with crisis		5	2	4	0		
04.99 Other and unspecified cardiac diseases				0	2	5	9
05.01 Chronic obstructive pulmonary disease					0	2	3
05.02 Asthma		0	0	0	1	1	1
06.01 Acute abdomen			1	1	2	2	2
06.02 Liver cirrhosis				0	0	0	0
07.01 Renal failure			0	0	0	1	1
08.01 Epilepsy		1	1	3	1	0	0
09.01 Ectopic pregnancy					0		
09.02 Abortion-related death					0		
09.03 Pregnancy-induced hypertension					1		
09.04 Obstetric hemorrhage					2		
09.06 Pregnancy-related sepsis					0		
09.07 Anemia of pregnancy					0		
09.99 Other and unspecified maternal CoD					0		
10.01 Prematurity	6						
10.02 Birth asphyxia	24						
10.03 Neonatal pneumonia	26						
10.04 Neonatal sepsis	6						
10.06 Congenital malformation	2	1	0				
10.99 Other and unspecified neonatal CoD	4						
12.01 Road traffic accident		0	1	7	5	1	1
12.03 Accidental fall				2	1	2	1
12.04 Accidental drowning and submersion		1	1	4	2		0
12.05 Accidental exposure to smoke fire & flame			1		0	0	0
12.06 Contact with venomous plant/animal						0	
12.07 Accidental poisoning & noxious substances						0	
12.08 Intentional self-harm				0	2	1	0
12.09 Assault				2	3	2	3
12.10 Exposure to force of nature			1	1			
12.99 Other and unspecified external CoD		0		0	0	0	0
98 Other and unspecified NCD					1	1	2
99 Indeterminate	5	12	9	8	7	8	10
VA not completed	25	8	23	13	17	14	10
Total	100	100	100	100	100	100	100

a WHO 2012 VA standard categories (26); CSMFs are rounded to nearest 1%, 0 representing a finite value <0.5%, and the blank cells are either impossibilities or cause/age combinations with no case assigned.

#### Infant deaths (*n*=286)

The top CODs in infants (excluding neonates) were pneumonia, HIV/AIDS, and malaria which accounted for 28, 21 and 12% of deaths, respectively. Five percent of deaths were attributed to sickle cell crisis while other COD categories totalled 14%. Twelve percent of deaths were indeterminate (Table 2). No sex-specific differences were noted with the exception of pneumonia, which was more common in female than male infants (Fig. 1).

#### Children 1–4 years old (*n*=302)

Malaria, HIV/AIDS, and pneumonia were the predominant CODs in this age group, causing 23, 18, and 12% of the childhood deaths, respectively, while diarrheal diseases, severe malnutrition, sickle cell with crisis, and road traffic accidents explained 3, 2, 2, and 1% of deaths, respectively. All other CODs accounted for 7% while a further 9% of deaths were indeterminate (Table 2). A slightly higher proportion of girls than boys died of pneumonia while no differences were noted between the sexes for death due to malaria (Fig. 1).

#### 5–14 years old (*n*=218)

Relatively fewer deaths were observed in this age group. The commonest CODs were HIV/AIDS related and malaria, which accounted for 23 and 13% of total deaths, respectively. Deaths due to pneumonia, road traffic accidents, meningitis/encephalitis, and sickle cell with crisis explained 8, 7, 5, and 4% of deaths, respectively. Eight percent of cases were indeterminate (Table 2). Unlike the previous age group, in this age group a higher proportion of boys appeared to die of pneumonia and external CODs than girls (Fig. 1).

#### 15–49 years old (*n*=1,068)

A third of deaths in this age group were attributed to HIV/AIDS and pulmonary tuberculosis. The majority of those who died of HIV/AIDS were either divorced or separated (60%). Road traffic accidents, assault, and stroke accounted for 5, 3, and 3%, respectively. Overall, 7% of deaths were indeterminate (Table 2). A higher proportion of women (29.1%) than men (12.2%) in this age group died of HIV/AIDS while more men (23.8%) than women (2%) died of external CODs (Fig. 1). A total of 62/565 deaths among women of reproductive age were due to maternal causes of which the two most common were obstetric hemorrhage (51.6%) and pregnancy-induced hypertension (25.8%).

#### 50–64 years old (*n*=729)

All neoplasms, pulmonary tuberculosis, HIV-/AIDS-related deaths and stroke were the predominant CODs among people of this age group, accounting for 16, 12, 12, and 10% of deaths, respectively. Other unspecified cardiac diseases explained 5% of deaths. Eight percent of the deaths were indeterminate (Table 2). More males (14.7%) than females (8.6%) died of pulmonary tuberculosis whereas more females (18.1%) than males (3%) died of HIV/AIDS. Women were more likely to die of stroke than men in this age group (Fig. 1).

#### Above 65 years old (*n*=1,513)

All neoplasms, stroke, and pulmonary tuberculosis predominated, accounting respectively for 15, 14 and 10% of total deaths. Cardiac diseases accounted for 10% of deaths and 10% were indeterminate (Table 2). A higher proportion of women (16.8%) than men (11.3%) died of stroke whereas in this age group men were more likely than women to die of pulmonary tuberculosis (Fig. 1).

The pattern of mortality as determined by the InterVA-4 model shows a high burden of infectious diseases, including HIV/AIDS, pneumonia, and pulmonary tuberculosis in the study population. These mortality patterns are consistent with existing knowledge on the burden of disease in many parts of sub-Saharan Africa.

## Discussion

The majority of previous studies that have reported CODs among rural populations in the developing world have used the physician certified verbal autopsy (PCVA) approach to COD assignment (27–33). A VA programme based on PCVA requires the involvement of a minimum of three clinicians with skills in the method. As a result, the approach is expensive, a distraction to skilled personnel who represent a scarce resource in less developed countries, and is a frequent bottleneck in the provision of timely data (3). Furthermore, the PCVA approach is vulnerable to bias from physician coders, who are prone to preconceptions about the common patterns of death in any given community that are not necessarily evidence based (34). Such considerations have justified the more recent development of automated approaches to VA coding (25, 35). One such method is the freely available, WHO-compliant, InterVA-4 model, a method that has recently been adopted by the INDEPTH Network of demographic surveillance sites as the only viable strategy currently available for the timely provision of comparable data from multiple populations (36). In the current study, we have used this method as the basis for assigning CODs in 4,460 subjects of all ages who were residents of the KHDSS area on the coast of Kenya, where we used the 2012 version of the InterVA-4 model to interpret VA data collected using the 2007 version of the WHO VA questionnaires.

In general, our COD estimates appear credible and conform to expected patterns. As anticipated, we found a slight excess of deaths among males (2,304; 52%) in comparison to females (2,156; 48%). This observation was consistent across all age groups individually with the exception of the 15–49 years age group where, predictably, we found an excess of deaths among women, predominantly from pregnancy-related causes and HIV/AIDS. Of particular interest, external CODs such as road traffic accidents were more commonly observed among men than women. Since Kenya has recently experienced a surge in the use of motorcycles as a mean of transport, and a majority of riders lack proper training, this is, perhaps, not surprising, but is nevertheless a matter of considerable concern. Similarly, palm wine tapping, a common socioeconomic activity for men within the KHDSS, can sometimes result in fatal accidental falls, especially when men climb the trees having sampled the product. In the elderly, 65+ age group, malignancies and stroke were key concerns, the latter being consistent with data from our previous study in which we reported hypertension and diabetes as major risk factors (12).

While in general the process of VA coding was straight forward, we did face some challenges with regard to the input and interpretation of our data. First, in comparison to that reported from previous studies (4, 37), a higher proportion of cases in our analysis were reported as ‘indeterminate’. The majority of these cases were associated with scanty signs and symptoms obtained from the respondents, a phenomenon that was particularly common where deaths were sudden or where subjects were found dead. Further research will be needed to establish more reliable methods for COD assignment in such cases. Second, HIV varies considerably from place to place and according to the model, it is necessary to specify whether HIV in the area is high, low or very low, corresponding to ratios of 1:100, 1:1,000 and 1:10,000 of all deaths, respectively (25). Since the prevalence of HIV infection in well-nourished and severely malnourished children within the KHDSS has been reported at 2 and 14%, respectively (38), we set the HIV variable within the model to ‘high’, as described in the InterVA-4 user guide (25). We suspect, however, that HIV may be overestimated as a cause of death among children within the current analysis, reflecting how difficult it can be to discriminate between HIV and malnutrition on the basis of clinical features alone. On the contrary, the high frequency of HIV/AIDS among adult women of reproductive age seems more plausible and is consistent with previous reports (12, 37). Third, because the InterVA-4 model is based on a more recent version of the WHO VA tools, we needed to map our input indicators from the format in which they were collected (on the 2007 version of the WHO VA questionnaire) onto the relevant indicators within the InterVA-4 model, a process that proved to be straight forward with regard to the majority of indicators. Finally, although in general the pattern of deaths reported in our study seems generally plausible, and is supported by our previous validation study conducted in adults within the same area (12), in the current study, COD interpretation has not been validated by any other method. In the future, we plan to undertake further work with this aim in mind.

A major advantage of computer-based coding methods such as InterVA-4 is their potential for providing standardized data across multiple sites and over long periods of time. For this to be viable, it will be important to agree on standardized methods for data input. For example, in our own data set some questions were missing from the WHO 2007 VA instrument but were present in the WHO 2012 instrument. However, these data were partially captured in the free-text sections of the VA form, for which we developed an automated search for the ‘keyword’ and mapped it onto the corresponding indicator. This was particularly important in capturing specific diagnoses such as SCD and specific forms of injury. While reviewing and modifying the questionnaires from time to time has certain advantages, it also leads to problems in the analysis and interpretation of longitudinal data. If the method is to be used successfully for the purpose of comparisons between multiple sites, collaborative discussion will be needed to agree on protocols for data input and interpretation.

## Conclusions

Our study suggests that both the WHO 2012 instrument and the InterVA-4 model are feasible tools to measure cause-specific mortality, which may potentially inform both health policy and program interventions in resource-limited settings. The model requires minimal time and labor resources, especially in comparison with the PCVA method. The current analysis, using InterVA-4, returned COD patterns that were generally credible at the population level and in subjects of all ages. While further work will be required to fine-tune and validate COD analysis using InterVA-4, particularly in children, we hope that our results will prove useful for informing health intervention policies both locally and internationally.
